# User Characteristics of a Low-Acuity Emergency Department Alternative for Low-Income Patients

**DOI:** 10.5811/westjem.2020.8.47970

**Published:** 2020-10-27

**Authors:** Sara W. Heinert, Melody Mumford, Sarah E. Kim, Muhammad M. Hossain, Michael L. Amashta, Maria A. Massey

**Affiliations:** * University of Illinois at Chicago, Department of Emergency Medicine, Chicago, Illinois; † University of Illinois at Chicago, Mile Square Health Center, Chicago, Illinois; ‡ University of Illinois at Chicago, College of Medicine, Chicago, Illinois

## Abstract

**Introduction:**

Emergency department (ED) use for healthcare that can be treated elsewhere is costly to the healthcare system. However, convenience settings such as urgent care centers (UCC) are generally inaccessible to low-income patients. Housing an UCC within a federally qualified health center (FQHC UCC) provides an accessible convenience setting for low-income patients. In 2014 a FQHC UCC opened two blocks from an ED in the same health system. Our goal was to compare characteristics, access to care, and utilization preferences for FQHC UCC and low-acuity ED patients through retrospective chart review and prospective surveying.

**Methods:**

We completed a retrospective chart review of all patients from March 1, 2018–March 1, 2019, and compared characteristics of low-acuity ED patients (N = 3,911) and FQHC UCC patients (N = 12,571). We also surveyed FQHC UCC patients (N = 201) and low-acuity ED patients (N = 198) from January–July 2019.

**Results:**

Half of FQHC UCC patients had private insurance. Of ED patients, 29% were aware of the FQHC UCC. Both groups had similar rates of primary care providers. The most common reason for choosing the ED was perceived severity, and for choosing a FQHC UCC was speed.

**Conclusion:**

These findings show similarities and differences between these two patient populations. Future research is needed to determine utilization patterns and in-depth reasons behind them. Interventions that help patients decide where to go for low-acuity care may create more utilization efficiency.

## INTRODUCTION

Emergency department (ED) visits in the United States increased by 14.8% from 2006 to 2014,^1^ with nearly 146 million total in 2016.^2^ Simultaneously, the proportion of ED visits that resulted in admissions decreased for all age groups.^3^ Because ED crowding can lead to poor patient outcomes,^4^–^8^ there is increased interest in diverting low-acuity patients to alternative sites for care. Urgent care centers (UCC) are one type of alternative site, or “convenience setting,” and tend to have availability beyond usual office hours and a broader scope of services than most primary care offices.^9^ UCCs also tend to have much lower average costs ($168)^10^ than the ED ($978–$2,259).^10^,^11^ However, these convenience settings are a topic of debate regarding their ability to replace EDs for care.

One study found that 13.7–27.1% of all ED visits could be treated at an UCC or retail clinic, which would result in a cost savings of approximately $4.4 billion per year.^12^ Others argue that patients who are more likely to use the ED for low-acuity conditions have little access to other types of care, including convenience settings.^13^ Convenience settings generally do not accept Medicaid^13^ and tend to be located in affluent areas where Medicaid patients do not live.^14^ Based on a national survey of 436 UCCs, 51% of patients had private insurance, 15% had Medicare, 12% were uninsured, and 10% had Medicaid.^9^

Comparatively, federally qualified health centers (FQHC) are much more accessible to a low-income patient population. FQHCs provide services regardless of patients’ ability to pay, charge for services on a sliding fee scale, and are located in underserved areas.^15^ Thus, the national FQHC patient/payor mix differs widely from the UCC payor mix, with 18% private insurance, 13% with Medicare or Medicare and Medicaid, 23% uninsured, and 49% with Medicaid.^16^ Housing an UCC within a FQHC allows for more accessibility for low-income patients than a freestanding UCC. This study compares characteristics of low-acuity ED and FQHC UCC patients. We also explored patient reasons and preferences behind their use of one site over the other. Housing an UCC within a FQHC is a unique concept, and as such is a largely unexplored topic.

## METHODS

### Study Setting

The University of Illinois (UI) Hospital ED is a 24-hour, state-funded academic facility with 31 licensed treatment spaces, within the 495-bed UI Hospital. In 2018 there were 48,835 ED visits, of which 18% were pediatric. Of total patients seen 1% were uninsured, 23% had Medicare, 43% had Medicaid, 32% had private insurance, and 1% had other insurance. The ED is not a trauma center and has a four-bed fast track area for low-acuity conditions that is staffed by nurse practitioners. Patients are triaged by a nurse using the Emergency Severity Index (ESI) on a scale of 1 to 5, which takes into account acuity and resource needs, and prioritizes incoming patients who need to be seen immediately.^17^ One is resuscitation (most urgent), 2 is emergent, 3 is urgent, 4 is less urgent, and 5 is non-urgent.^17^

Mile Square FQHC, which is part of the same health system as the ED,^18^ has predominantly minority patients with public or no insurance, many of whose incomes are below the federal poverty level.^18^ In addition to clinic services, the main location of the FQHC – approximately two blocks from the ED – houses an UCC, which opened in March 2014. The FQHC UCC is advertised as “Less wait. Less cost. Many of the same services as the E.R.”^19^ It treats injuries that require radiographs, simple lacerations, cold/flu symptoms, and other minor illnesses and injuries, and is open beyond normal business hours, including on weekends and holidays.^19^ The FQHC UCC is staffed by physicians and midlevel providers; it has 10 rooms with two additional rooms for triage.

The FQHC UCC has seen more patients each year, with 7881 in 2014 and 16,608 in 2018. From 2014 to 2018 the proportion of ED visits categorized as ESI 2 (second highest severity) increased from 12.0% to 17.3%, ESI 4 (second lowest severity) visits decreased from 32.0% to 25.4%, and hospitalizations from the ED increased from 26.5% to 28.5%. The State of Illinois expanded Medicaid in January 2014, so the number of people with insurance also increased during this time.

**Population Health Research Capsule**

What do we already know about this issue?

*Emergency department (ED) use for care that can be treated elsewhere is costly, but settings such as urgent care centers (UCC) are generally inaccessible to low-income patients*.

What was the research question?

What are the characteristics of federally qualified health center (FQHC) UCC patients compared to low-acuity ED patients?

What was the major finding of the study?

*Half of FQHC UCC patients had private insurance. Groups had similar access to care. Most common reason for ED use was severity and for FQHC UCC speed*.

How does this improve population health?

*Helping patients decide where to seek low-acuity care may create more use efficiency, especially for the low-income patient population that FQHCs support*.

There are similarities in access for both sites. Since they are two blocks apart, there are no geographic differences between the site locations. Additionally, because both sites are within the same healthcare enterprise, patients who prefer the continuity of being seen within the same system can be seen at either site, and providers can access patient health information across both sites. Because it is within a FQHC, the FQHC UCC cares for patients regardless of insurance status or ability to pay. As a result, the FQHC UCC may be more substitutive of low-acuity ED visits than other UCCs for low-income patients.

### Procedure and Participants

To study the entire patient population for each site, we accessed electronic health record (EHR) data (Cerner Corporation, Kansas City, MO) for visits from March 1, 2018–March 1, 2019, for two groups of adult patients (18 years and older): 1) ED patients with ESI of 4 or 5; and 2) FQHC UCC patients. We only included ED patients who arrived when the FQHC UCC was open. From March 1–December 31, 2018, the FQHC UCC was open from 12–8 pm on weekdays and 10 am–6 pm on weekends and holidays. These hours changed on January 1, 2019, to 8 am–7 pm on weekdays and 10 am–5:30 pm on weekends and holidays.

To obtain more detailed information from FQHC UCC and low-acuity ED patients, we also conducted a survey at each site about demographics, the current day’s visit, access to care, healthcare utilization and satisfaction, and reasons for choosing one site over the other for current day’s care. Survey questions were pilot tested to ensure patient comprehension and appropriate survey length.

At both locations, patients were approached between 9 am–5 pm, Monday–Friday, and surveys were available in English and Spanish. Research assistants (RA) confirmed eligibility by reviewing the patient list on FirstNet Organizer, the ED patient board, and with some provider assistance. Eligible ED patients were adults with ESI of 4 or 5, and eligible FQHC UCC patients were all adults. After approaching the potential participant using a recruitment script, the RA gave the ED patients five minutes to think about whether they wanted to participate and then returned. (The five-minute wait was not required due to the fast pace of the UCC).

After obtaining written consent, the RA read the survey questions aloud and recorded responses, in order to avoid literacy barriers. The surveys took approximately 10 minutes to complete. Upon completion, surveys were entered in REDCap (Center for Clinical and Translational Science (CCTS) UL1TR002003) using double-data entry. To determine whether there were significant differences between patients who presented to the ED with low-acuity needs and patients who presented to the FQHC UCC, we ran t-tests for continuous variables and chi-square and Fisher’s exact tests were run for categorical variables. We completed all data cleaning and analysis using Stata Version SE 15 (StataCorp LP, College Station, TX). The study was approved by the Institutional Review Board of the University of Illinois at Chicago.

## RESULTS

### Chart Review Results

Table 1 shows a comparison of demographic characteristics between FQHC UCC patients and low-acuity ED patients for the EHR review over the period of March 1, 2018–March 1, 2019. The proportions of female, White, and private insurance patients were significantly higher for the FQHC UCC. The ED had significantly higher proportions of Medicaid, Medicare, uninsured, and other insurance patients compared to the FQHC UCC. More ED patients than FQHC UCC patients were seen outside of regular business hours.

### Survey Results

#### Demographics

Looking across site for survey participants, FQHC UCC patients tended to have more education; the site had higher proportions of patients with full-time employment, and lower proportions of students and unemployed patients than those in the ED. There were more FQHC UCC survey participants who lived in the immediate ZIP codes of the ED and FQHC UCC, but not at a significant difference than survey participants in the ED.

#### Current Day’s Visit

Table 1 also shows that more ED patients reported excellent health (17.7%) than FQHC UCC patients (4.5%). A small proportion of patients (4.6%) arrived by ambulance, whereas that mode of arrival was not available for FQHC UCC patients. More ED patients arrived by public transportation compared to FQHC UCC patients (24.2% and 18.4%, respectively), and more ED patients than FQHC UCC patients received a ride from family/friends (31.3% and 12.4%, respectively). FQHC UCC patients had more than double the proportion of patients who drove themselves in their own vehicles (53.2%), compared to ED patients (25.8%).

#### Access to Care/Healthcare Utilization and Satisfaction

Table 2 shows that patients at both sites had similar responses to how often it was easy to get care, tests, or treatment in the prior six months, with the majority (68.2% for ED and 64.7% for FQHC UCC) responding with “always.” For patients who did not respond “always,” the most common reason for both groups to not be able to get care was because the wait took too long (32.8% for ED, and 50.0% for FQHC UCC). While not significant, there were higher proportions of ED than FQHC UCC patients with socioeconomic-related issues such as inability to get care because they could not afford it, their health plan would not cover it, they could not get transportation to the doctor’s office, or could not take time off/get child care.

When asked about what kind of place you go to most often for your medical care, a much higher proportion of ED patients selected the ED (18.9%) than FQHC UCC patients (3.5%), and a higher proportion of FQHC UCC patients selected “other place” (19.4%) compared to ED patients (0.0%). Presumably, this “other place” is the FQHC UCC, although it was not explicitly asked in the survey.

There was not a significant difference between the percent of patients in each group with a primary care provider (PCP), the length of time with their current PCP, and their satisfaction with their PCP. Approximately 35% of ED patients surveyed had ever been to a FQHC. ED patients who had used a FQHC before had significantly more FQHC visits in the prior year (2.6) than FQHC UCC patients (1.4) with no significant difference in satisfaction. About 36% of ED patients said they were not sure how available FQHCs were in their neighborhood, and 18.3% said that they were not at all available.

Forty-two percent of low-acuity ED patients had been to an UCC before. Of those patients who had been to an UCC, ED patients had used one an average of 1.3 times in the prior year, which was significantly less than FQHC UCC patients who had used one 2.4 times. The mean satisfaction rating of UCCs for ED patients was lower (7.9) than for FQHC UCC patients (9.0). Half of low-acuity ED patients said UCCs were not at all available in their neighborhood or they were unsure about availability, and 18% said they were very available. For FQHC UCC patients, 40% said they were unsure or not at all available, and 41% said they were very available. For ED utilization and satisfaction, the low-acuity ED group had significantly more ED visits on average in the prior year (2.5), compared to the FQHC UCC group (1.1). Of those who used the ED, the low-acuity ED group had higher satisfaction with their experience (8.4) than the FQHC UCC group (6.0).

#### Unique questions for ED patients

Table 3 shows the questions that were only asked of the low-acuity ED patients. The top three reasons for choosing the ED were the following: problem was too serious for the doctor’s office (44%); doctor’s office/clinic was open but could not get an appointment (15%); and patient gets most care from the ED (8%). Approximately 17% had called their PCP prior to coming to the ED. Twenty-nine percent knew that there was an UCC at the FQHC, and 21% had ever used the FQHC UCC.

#### Unique Questions For FQHC UCC Patients

Table 4 shows questions that were only asked of the FQHC UCC patients. The top three reasons for choosing the FQHC UCC instead of the ED were the following: faster/more efficient/less wait (36%); perceived their medical issue to be less urgent (31%); and less cost (12%). When asked how they heard about FQHC UCC, the top three places were from a medical professional (doctor, clinic, or hospital) (33%), family/relatives (21%), and the Internet (15%).

## DISCUSSION

There were several interesting findings from this study. The chart review and survey showed that approximately half of FQHC UCC patients had private insurance, and the FQHC UCC had lower proportions of Medicaid and uninsured patients than the ED. This was surprising, given that the private insurance population has more available alternatives to the ED than Medicaid and uninsured patients. Since a FQHC UCC is unique, Medicaid and uninsured patients may be hesitant to visit the FQHC UCC if they know that they would have to pay higher costs at freestanding UCCs or be unaware of how UCCs work if they have never used one before. Despite these differences, self-reported access to care was similar across both sites with both groups having similar proportions of patients with PCPs, and similar proportions of patients who “usually” or “always” got the care or treatment they needed.

A higher proportion of ED patients said that UCCs were not available or they were unsure if they were available in their neighborhood compared to FQHC UCC patients, and more FQHC UCC patients said that UCCs were very available in their neighborhood than ED patients. It is unclear whether ED patients truly have fewer UCCs in their neighborhood, or whether they were unaware of UCCs in their neighborhood because they were unsure of their purpose or because they did not see them as a site of care that was accessible for them. Likewise, only one-third of ED survey participants had been to a FQHC (despite one being two blocks from the ED where they presented for care), and about one-quarter were aware of the FQHC UCC. These findings suggest that basic education for ED patients with low-acuity conditions on the purpose and benefits of FQHCs and UCCs might be an appropriate first step in having patients understand their ability to use such healthcare sites. Additionally, future work could compare the payor mix of the FQHC UCC patients and other nearby freestanding UCCs.

Research has shown that low-income patients tend to prefer hospital care over primary care,^20^ which may help explain the larger proportion of Medicaid patients in the ED. In our survey, we found that satisfaction with the ED was significantly higher for ED patients than FQHC UCC patients, and satisfaction with an UCC (for those who have used them previously) was significantly higher for FQHC UCC survey participants than ED survey participants. Also, it may be beneficial to create future interventions that share the positive reasons that FQHC UCC patients gave for using the FQHC UCC with ED low-acuity patients.

This study is a first step in exploring utilization of a FQHC UCC compared to low-acuity ED visits at a nearby ED. Future work should look at changes in utilization patterns for low-acuity conditions when the FQHC UCC opened in 2014, and determining characteristics of patients who shifted their low-acuity care from the ED to the FQHC UCC. Prior literature has suggested a shift away from EDs for certain low-acuity conditions when new UCCs open nearby for non-Medicaid patients, as Medicaid patients do not have access to UCCs.^21^ Another study has shown that after an ED patient had his or her first visit to an UCC, his or her non-emergent ED use decreased by 48%.^22^ Hence, we would want to explore whether these trends are present with the FQHC UCC, which is more accessible to Medicaid and uninsured patients than regular UCCs.

Our findings concur with qualitative interviews where Medicaid enrollees presented to the ED for non-urgent needs because they believed their condition was too serious for the PCP and that the ED provided more comprehensive services.^23^ Authors of this study stressed the importance of improving patients’ understanding of where to seek care, and look beyond logistical and access-related concerns.^23^ These conclusions support our study, where logistically and geographically, the ED and FQHC UCC were very similar, and both groups had similar access to care availability. Furthermore, the fact that our study was a brief survey only begins to touch on patient barriers and facilitators to using the FQHC UCC for low-acuity conditions, as well as decision-making and preferences. Future in-depth qualitative work with low-acuity ED and FQHC UCC patients can tell a more complete story.

## LIMITATIONS

This study had several limitations. First, our eligibility criteria for low-acuity ED patients was based on ESI, which can be subjective, as it is determined by humans and is prone to human error and opinions. However, it was our best proxy for the patient’s acuity level in the ED. There are several ways to look at low-acuity ED visits, and future research may incorporate additional methods, such as the NYU Algorithm, which incorporates the probability that given the ED patient’s diagnosis, he or she could have been seen elsewhere.^24^ Second, the proportions were very similar between survey participants and all low-acuity ED and FQHC UCC patients (Table 1), which suggests that the survey sample was representative of these patient populations. However, the one area where proportions were different was insurance type. There was a much higher proportion of private insurance patients for survey participants in the ED, compared to the chart review of all low-acuity ED patients. Likewise, being uninsured was much lower for ED survey participants than for all low-acuity ED visits in the chart review. As a result, patients with private insurance may have been more likely to complete the survey than uninsured patients and/or there may have been more private insurance patients than uninsured patients in the ED during regular business hours, when the survey was being administered. This is a limitation of the study and our results might not fully represent the experiences of all low-acuity ED patients. Additionally, the survey did not ask about whether FQHC UCC patients knew about the nearby ED, which would have helped to determine the degree of overlap between the two groups and general awareness of the ED. Lastly, the study only included one ED and one FQHC UCC. More work should be done in similar models to determine whether these findings persist with additional sites.

## CONCLUSION

While these findings provide a starting point for similarities and differences between these two patient groups, future research is needed to determine utilization patterns for these patients, as well as more in-depth reasons behind these patterns. The concept of a federally qualified health center urgent care center is new and lacks research. Expansion of the model may provide more accessible and cost-saving healthcare options for low-income patients.

## Figures and Tables

**Table 1:** Emergency department patients with Emergency Severity Index (ESI) 4 or 5 survey respondents, and federally qualified health center urgent care center patient survey respondents: demographics and the current day’s visit.

	Chart review findings			Survey findings		

	ED (N=3,911)	FQHC UCC (N=12,571)	P-value	ED (N=198)	FQHC UCC (N=201)	P-value
Age, mean (SD)	38.0 (15.8)	39.0 (14.3)	0.001	38.7 (15.9)	38.7 (14.2)	0.984
% Female	60.2	71.0	<0.001	66.0	69.7	0.434
Race^a^						
% White	9.4	12.3	<0.001	7.9	20.9	0.002
% Black	58.9	57.2		68.6	54.7	
% Asian	2.8	3.0		3.1	4.0	
% Other	29.0	27.6		20.4	20.4	
Ethnicity						
% Hispanic	24.0	24.3	0.706	20.2	22.9	0.515
Insurance type^a^						
% Medicaid	45.1	36.8	<0.001	40.8	35.8	0.368
% Medicare	6.0	4.6		9.2	6.5	
% Uninsured	22.0	12.6		9.2	8.0	
% Private insurance	24.1	45.2		44.9	52.7	
% Other^b^	2.9	0.7		0.0	0.0	
% Patients living in ZIP codes that encompass the medical campus (ED and FQHC UCC) (60608 & 60612)	17.8	20.3	0.001	17.2	22.4	0.602
% Patients seen on weekend	26.0	19.9	<0.001			
% Patients seen during business hours (8 AM-5 PM, Monday-Friday)	51.0	63.0	<0.001			
Employment status^a^						
% Full time				41.9	54.7	0.030
% Part time				15.2	14.4	
% Unemployed				24.8	18.4	
% Student				13.1	5.0	
% Other^c^				7.1	9.0	
Highest level of education completed						
% 8th grade or less				1.5	1.0	0.001
% Some high school, but did not graduate				11.6	6.5	
% High school graduate or GED				30.8	23.9	
% Some college or 2-year degree or trade school grad				37.4	30.9	
% 4-year college graduate				12.1	19.4	
% More than 4-year college graduate				6.6	18.4	
Self-reported health status						
% Excellent				17.7	4.5	0.001
% Very good				24.2	31.0	
% Good				34.3	36.5	
% Fair				19.7	23.5	
% Poor				4.0	4.5	
Mode of transportation for the current day’s health visit^d^						
% Ambulance				4.6	0.0	<0.001
% Public transportation				24.2	18.4	
% Taxi or ride share				7.6	8.5	
% Drove self in own vehicle				25.8	53.2	
% Received ride from family/friend				31.3	12.4	
% Medicab				0.5	0.0	
% Walked				6.1	7.5	

a More than one response could be selected.

b Other insurance includes those that did not fit in other categories, such as Worker’s Compensation and Civilian Health and Medical Program of the Uniformed Services (CHAMPUS).

c Includes self-employed (n=4), in between jobs (n=1), disability (n=9), retired (n=17), and other not specified (n=1).

d “% Ambulance” has been included in the table but was excluded from statistical analysis between the two groups because FQHC UCC patients do not have the ability to arrive by ambulance.

Note: For the chart review, analysis was conducted at the visit level, so it was possible for the same patient to be included more than once if they presented multiple times or would be included in both groups if they were seen at both sites.

*ED*, emergency department; *FQHC UCC*, federally qualified health center urgent care center; *GED*, General Educational Development.

**Table 2:** Survey findings: Emergency department patients with Emergency Severity Index 4 or 5 survey respondents and federally qualified health center urgent care center patient survey respondents–access to care/healthcare utilization and satisfaction.

	ED (N=198)	FQHC UCC (N=201)	P-value
In the last 6 months, how often was it easy to get care, test, or treatment you needed?^a^			
% Never	3.0	3.0	0.791
% Sometimes	14.1	13.9	
% Usually	14.7	18.4	
% Always	68.2	64.7	
(If Never, Sometimes, or Usually:) What is the main reason you were not able to get medical care, tests, or treatments that you or a doctor believed necessary?^a^ (ED n=58, FQHC UCC n=62)			
% Couldn’t afford	12.1	9.7	0.481
% My health plan wouldn’t approve/cover/pay for care	19.0	12.9	
% Doctor refused to accept my insurance	8.6	6.5	
% Doctor doesn’t speak my language	0.0	0.0	
% Couldn’t get transportation to doctor’s office	6.9	1.6	
% Couldn’t take time off work/get child care	19.0	16.1	
% Didn’t know where to go to get care	1.7	3.2	
% The wait took too long	32.8	50.0	
What kind of place do you go to most often for your medical care?^a^			
% Clinic or health center	42.4	47.8	<0.001
% Doctor’s office or HMO	32.1	26.9	
% Hospital ED	18.9	3.5	
% Hospital outpatient department	5.6	1.5	
% Other place	0.0	19.4	
% Don’t go to one place most often	0.0	1.0	
% There is no place visited often for medical care	1.0	0.0	
Primary care provider (PCP)			
% with a PCP	75.8	71.6	0.681
Of those with a PCP, length of time with current PCP (in years)	26.4 (13.2)	25.6 (1.1)	0.632
Of those with a PCP, satisfaction with PCP (1=least, 10=best)	8.7 (2.2)	8.6 (1.8)	0.786
Federally qualified health center (FQHC)			
% who have ever been to a FQHC	34.9	N/A	N/A
Frequency of usage in past year, mean (SD)	2.6 (4.7)	1.4 (2.2)	0.003
Satisfaction with FQHC experience (1=least, 10=best)	8.1 (2.8)	8.7 (1.6)	0.111
How available are FQHCs in your neighborhood?			
% Not at all available	18.3	N/A	N/A
% Rarely available	6.6		
% Somewhat available	19.8		
% Very available	19.8		
% Unsure	35.5		
Urgent care center			
% who have ever been to an urgent care center	41.9	N/A	N/A
Frequency of usage in the past year	1.3 (1.4)	2.4 (2.5)	<0.001
Satisfaction with urgent care center experience (1=least, 10-=best)	7.9 (2.5)	9.0 (1.7)	<0.001
How available are urgent care centers in your neighborhood?			
% Not at all available	19.9	15.9	<0.001
% Rarely available	10.2	4.0	
% Somewhat available	21.9	14.9	
% Very available	17.9	41.3	
% Unsure	30.1	23.9	
Emergency department			
Frequency of usage in the past year	2.5 (2.3)	1.1 (2.5)	<0.001
Of those who used the ED, satisfaction with ED experience (1=least, 10=best)	8.4 (2.2)	6.0 (3.2)	<0.001

a Question Source: Nationwide Adult Medicaid Consumer Assessment of Healthcare Providers and Systems questionnaire, Medicaid.gov. Centers for Medicare & Medicaid Services. https://www.medicaid.gov/medicaid/quality-of-care/performance-measurement/adult-cahps/index.html

*ED*, emergency department; *FQHC UCC*, federally qualified health center urgent care center; *HMO*, health maintenance organization; *SD*, standard deviation.

**Table 3:** Unique questions for emergency department patients with Emergency Severity Index score 4 or 5 survey respondents (N = 198).

Main reason for emergency department visit today	
% Problem was too serious for the doctor’s office	43.9
% Doctor’s office/clinic was open, but could not get an appointment	15.2
% Get most of my care at the emergency department	8.1
% Didn’t have a doctor	6.6
% Doctor’s office/clinic was not open	5.6
% Other (n=41)	20.7
Told to go or brought to ED by medical professional (n=12)	
ED is more efficient/quick than other healthcare options (n=8)	
Preference for ED over other healthcare providers (n=4)	
Location/convenience (n=4)	
Connection to the hospital (self or family member is employee or existing patient) (n=3)	
Went to ED without thinking about other options (n=3)	
Lack of experience with the healthcare system (n=2)	
Needed x-ray (n=2)	
Wanted to be extra careful (n=1)	
No transportation to doctor’s office (n=1)	
Insurance card expired so went to ED (n=1)	

% who called PCP prior to coming to the ED	16.7
% who know that there is an urgent care center in the FQHC	28.8
% who have used the urgent care center at the FQHC	21.2

*ED*, emergency department; *PCP*, primary care provider; *FQHC*, federally qualified health center.

**Table 4:** Unique questions for federally qualified health center urgent care center patient survey respondents (N = 201).

Why did you decide to use Mile Square instead of the ED today?	
*(Multiple reasons could be mentioned by one person) Top 5 Reasons Mentioned:*	

Faster/more efficient/less wait (n=72)	
Less urgent issue (n=63)	
Cost/cheaper (n=25)	
Referred by medical professional (n=13)	
Familiarity/comes to this FQHC regularly/been to this FQHC urgent care before (n=12)	

How did you hear about the FQHC urgent care center?	

% Family/relatives	21.2
% Online website	15.3
% Friend	5.9
% Other	57.6
Doctor/clinic/hospital (n=66)	
Drove by/saw it/lives close by (n=15)	
Work (n=10)	
Insurance (n=10)	
Has been a patient at this FQHC before (n=10)	
“Always knew” (n=2)	
General word of mouth (n=2)	
“Visiting” (n=1)	
“Myself” (n=1)	
Community based organization (n=1)	

*ED*, emergency department; *FQHC*, federally qualified health center.
